# Apportioned voting

**DOI:** 10.1371/journal.pone.0317580

**Published:** 2025-03-03

**Authors:** Lloyd W. Koenig

**Affiliations:** Retired, Annandale, Virginia, United States of America; University of Siena, ITALY

## Abstract

The paper introduces a new electoral system, based on proportional representation, called *apportioned voting* because each vote is apportioned among the candidates. Apportioned voting can be thought of as an enhanced and generalized hybrid of cumulative voting and single transferable vote (also known as proportional ranked-choice voting). Apportioned voting can efficiently handle government and corporate elections with large numbers of voters, positions to fill, and candidates. The paper provides a detailed description of apportioned voting, illustrative examples of apportioned voting’s election performance, and the Octave scripts used to implement apportioned voting and compute the example results.

## Introduction

The paper presents a new electoral system, based on proportional representation, called apportioned voting (APV) [1–3]. It is called apportioned voting because each vote is apportioned among the candidates. Some features of APV resemble features of cumulative voting (CV) [4–6], and others resemble features of single transferable vote (STV) (also known as proportional ranked-choice voting) [7–10].

Below is an introduction to APV that does not use mathematical notation, with links to the corresponding instructions of the more detailed and mathematical description of APV in Subsection APV description of the next section. Subsection Notation reference describes the mathematical notation used in Subsection APV description.

Under both CV and APV, voters complete their ballots by assigning to each candidate a nonnegative number representing the voter’s self-assessed level of support for, or numerical rating of, the candidate relative to the other candidates [4–6]. The completed ballots represent nonnegative functions on the set of candidates. Conceptually, a voter’s completed APV ballot can represent any nonnegative function on the set of candidates. Voters have control over the complexity of their voting task by their choice of a numerical candidate-rating system. The simplest numerical rating system is *binary approval voting*. The voter assigns one to each candidate whose election would be acceptable and zero to each candidate whose election would be unacceptable to the voter. Note that APV encourages the participation of political parties in elections by permitting voters to give the same, or nearly the same, level of support to candidates who represent the same political party. See an example of a possible APV ballot system in Subsection Discussion that permits the allocation of support to candidates individually and by party.

Under APV and STV, the candidate-elimination sequence determines the election result. Each vote influences the candidate-elimination sequence. APV maximizes this influence, consistent with the vote’s expression of the voter’s relative preferences for one candidate over another, by, in each iteration, subtracting the smallest value in the vote from every value in the vote (APV Instruction 1.2) and, then, normalizing the vote so that its entries sum to one (APV Instruction 1.3). This is done because voters would not want the effectiveness of their votes diminished by the allocation of nonzero support to their least favored candidates.

APV and CV weight their voter inputs [4–6]. They can weight them equally, as is common in government elections, or unequally, as is common in corporate elections [4–6]. APV’s weights are not voter inputs, but are input separately. APV uses the products of each normalized vote and its weight when computing each candidate’s weighted-vote total (APV Instructions 1.4 and 1.5.6).

APV resembles STV with respect to the conditions under which candidate support is reallocated, candidates are eliminated, and candidates are elected [7–10].

When the number of candidates is greater than the number of positions to be filled by the election, both APV and STV use a *threshold* or *quota* to identify elected candidates [7–10]. APV uses as its threshold the sum of the weights used to weight the votes cast in the election divided by the sum of one plus the number of positions to be filled by the election. APV identifies a candidate as *elected* when either the number of candidates equals the number of open seats (APV Instructions 1 and 2) or the candidate’s weighted-vote total is *not deficient* (greater than or equal to the threshold) and at least one candidate’s weighted-vote total is *excessive* (greater than the threshold) (APV Instructions 1.5 and 1.5.1).

While at least one candidate’s weighted-vote total is excessive (APV Instruction 1.5), APV identifies elected candidates (APV Instruction 1.5.1). If the number of candidates identified as elected exceeds the number of positions to be filled by the election, then computational rounding errors have occurred at the limit of the computer’s precision. APV identifies and corrects the errors (APV Instruction 1.5.2) and continues from APV Instruction 1.6. If the number of candidates identified as elected equals the number of positions to be filled by the election, then APV returns these candidates as the election winners and exits (APV Instruction 1.5.3). Otherwise, to help fill the remaining unfilled positions, APV redistributes (transfers) the amount by which the weighted-vote total of each elected candidate exceeds the threshold to the candidates whose weighted-vote totals are *deficient* (less than the threshold) as follows:

- For each candidate whose weighted-vote total is excessive, APV removes the excess by multiplying every vote’s level of support for the candidate by the threshold divided by the candidate’s weighted-vote total (APV Instruction 1.5.4). The weighted-vote totals of these candidates now equal the threshold.
- APV transfers the above excess amounts to the candidates with weighted-vote totals below the threshold as follows: For each vote, APV normalizes the vote on the set of candidates whose weighted-vote totals are below the threshold to sum to one on the set; then, multiplies the normalized portion of the vote by one minus the sum of the remaining portion of the vote (APV Instruction 1.5.5). Every vote is now normalized to sum to one.
- APV, then, updates the weighted-vote totals of the candidates (APV Instruction 1.5.6) and returns to the beginning of the while loop (APV Instruction 1.5).

At the completion of the above while loop, no candidate weighted-vote total is excessive. APV finds the largest candidate weighted-vote total that is greater than the sum of the weighted-vote totals of all candidates with smaller weighted-vote totals (APV Instruction 1.6.1), and, if any exist, then eliminates these candidates as defeated (APV Instruction 1.6.2), otherwise, APV randomly selects and eliminates one of the multiple candidates with the smallest weighted-vote total (APV Instruction 1.6.3). Then APV begins again with the set of remaining candidates as the new candidate set (APV Instruction 1).

APV treats votes as numerical voter ratings of the candidates and can accept any nonnegative voter ratings of the candidates as input. When voting sincerely under apportioned voting, a voter assigns to each candidate on the voter’s ballot the value of the candidate to the voter if elected relative to the other candidates on the ballot.

Reference [11] proves that: “It is NP-complete to determine whether there exists a (possibly insincere) preference that will elect a favored candidate, even in an election for a single seat. Thus, strategic voting under STV is qualitatively more difficult than under other commonly-used voting schemes. Furthermore, this resistance to manipulation is inherent to STV and does not depend on hopeful extraneous assumptions like the presumed difficulty of learning the preferences of the other voters. We also prove that it is NP-complete to recognize when an STV election violates monotonicity.” These results appear to apply to APV as well because

- The algorithms of APV and STV are similar. Both use successive candidate elimination to obtain their election results. Reference [11] identifies successive candidate elimination as the reason that strategic voting and non-monotonicity are NP-complete under STV.
- APV’s voter inputs can make APV behave like STV (see Subsection Discussion).

APV works at the candidate, not party, level. Nonetheless, APV’s process becomes a divisor method for apportioning seats among political parties when the election is multi-seat, multiparty, and partisan, and each party has enough candidates to fill all seats. The divisor is APV’s threshold, *t*_0_, and *partisan* means that every candidate belongs to exactly one party and each vote cast must support and only support one-party’s candidates [12].

The partisan structure of the election determines each party’s “proportional” minimum allotment of seats as follows (using notation from Subsection Notation reference and the proof of STV’s “proportional” representation in Reference [9] as a model):

Suppose that, in a partisan election,

- The number of open seats is *n*.
- For each candidate, *c*, wvt(*c*) is candidate *c*’s initial weighted-vote total. Then the sum of wvt(*c*) over all candidates, *c*, is (*n* + 1) * *t*_0_ by the definition of *t*_0_.
- For each political party, *p*,
  - C(*p*) is the set of candidates representing party *p*.
  - Party *p*’s initial weighted-vote total, wvt(*p*), is the sum of wvt(*c*) over all candidates, *c*, in C(*p*).

Then, for each political party, *p*,

- The minimum number of elected candidates in C(*p*) is $\text{j}\left(p\right)=\left\lceil \frac{\text{wvt}\left(p\right)}{t_{0}}-1\right\rceil$, where, for every real number, *r*, ⌈*r*⌉ is the smallest integer greater than or equal to *r*, and it is not possible for APV to identify the last *available* (not identified as elected or defeated) candidate in C(*p*) as defeated until j(*p*) candidates in C(*p*) have been identified as elected because
  - $\text{j}\left(p\right)<\frac{\text{wvt}\left(p\right)}{t_{0}}\leq \text{j}\left(p\right)+1$ and
  - Party *p*’s votes give nonzero support to and only to the available and elected candidates in C(*p*) as long as C(*p*) contains available candidates. [Note that, when C(*p*) does not contain available candidates and a seat is still open, party *p*’s votes can give nonzero support to available and elected candidates of parties other than *p* by transferring excess support from elected candidates supported by these votes to the available candidates of parties other than *p*.]
- The maximum number of elected candidates not in C(*p*) is *n* − j(*p*) because
  - The sum of wvt(*c*) over all candidates, *c*, not in C(*p*) is (*n* + 1) * *t*_0_ − wvt(*p*) by the definition of *t*_0_, and
  - $n-\text{j}\left(p\right)\leq n+1-\frac{\text{wvt}\left(p\right)}{t_{0}}<n+1-\text{j}\left(p\right)$.
- Since APV identifies exactly *n* elected candidates, APV will identify at least j(*p*), but no more than j(*p*) + *n* − *jsum*, elected candidates in C(*p*), where *jsum* is the sum of j(*q*) over all political parties, *q*. How APV determines C(*p*)’s final allotment of seats is not fully understood. (See [12] and Subsection Example 1: proportional representation.)

Subsection APV description of the next section provides a detailed mathematical description of APV. Subsection Notation reference describes the mathematical notation used in Subsection APV description. APV was coded, tested, and refined, in the Octave scripting language [13]. Octave scripts that implement APV are given in Subsection Octave scripts along with the scripts used to run the examples of APV’s election performance given in Subsection Apportioned-voting examples of Section Results and discussion [13]. Subsection Discussion of Section Results and discussion discusses features, potential applications, and benefits of APV and gives an example of a possible ballot system that permits the allocation of support to candidates individually and by party.

## Materials and methods

Subsection APV description gives a detailed mathematical description of APV.

Subsection Notation reference describes the mathematical notation used in Subsection APV description.

Subsection Octave scripts gives listings, in the Octave scripting language, of the scripts for the functions and user-defined object class, ptr, used to implement APV, and the scripts used to run the election examples given in Subsection Apportioned-voting examples of Section Results and discussion [13].

### APV description

APV may be described as follows (refer to Subsection Notation reference for descriptions of the mathematical notation):

Voters complete their ballots by assigning to each candidate a nonnegative number representing the voter’s self-assessed level of support for or rating of the candidate relative to the other candidates. If a voter fails to assign a value to a candidate, the value should default to zero so that voters do not have to assign a value to every candidate on the ballot. Conceptually, a voter’s completed ballot may represent any nonnegative function on the set of candidates. (See an example of a possible APV ballot system in Subsection Discussion that permits the allocation of support to candidates individually and by party.)

Voters have control over the complexity of their voting task by their choice of a numerical rating system, with binary approval voting being the simplest. Note that APV encourages the participation of political parties in elections by permitting voters to give the same, or nearly the same, level of support to candidates who represent the same political party.

APV stores and processes votes as probability mass functions (pmfs) on sets of candidates. A *pmf* on a set of candidates is nonnegative, sums to one over the set, and represents each vote’s allocation of support among the candidates in the set. APV automatically converts votes into their corresponding pmfs using function pmf (see Subsection Notation reference for a description of function pmf).

APV returns the set of elected candidates as a function of the following inputs:

1. The number, *n* > 0, of open positions to be filled by the election.
2. A vector, **c**, of unique positive integers identifying the candidates from whom the *n* open positions may be filled. (|**c**| must be at least *n*. If |**c**| equals *n*, APV returns **c** as the set of elected candidates.)
3. A matrix, **V**, of the votes cast in the election, where, for each vote (row) index, *x*, and candidate (column) index, *y*, **V**(*x*, *y*) is the nonnegative number (relative level of support or rating) that the *x* th voter specified for the *y* th candidate on the voter’s ballot. The elements of **c** must be column indices of **V**.
4. A row vector, **w**, of positive numbers (weights) such that |**w**| equals the number of rows that **V** has and, for each row index, *x*, of **V**, **w**(*x*) is the weight given to the *x* th vote when computing each candidate’s weighted-vote total.

APV uses a *threshold* value for candidate weighted-vote totals to identify elected candidates. The threshold is *t*_0_ = ‖**w**‖_1_/(*n* + 1), where ‖**w**‖_1_ is the sum of the weight values in vector **w**.

APV selects candidates to fill the open positions as follows:

1 While the number, |**c**|, of candidates represented in **c** exceeds the number, *n*, of open positions, do the following:
  1.1 Set **F** equal to the restriction, **V**(:, **c**), of the votes-cast matrix, **V**, to the candidates represented in **c**. Note that each column of **F** represents the same candidate as the corresponding element of **c**.
  1.2 Subtract the smallest value in each row of **F** from every value in the row.
  1.3 Convert the rows of **F** to their corresponding pmf vectors by setting **F** equal to *pmf*(**F**).
  1.4 Set **f** equal to the row vector **w** * **F**. Each element of **f** is the weighted-vote total of the candidate represented by the corresponding element of **c**.
  1.5 While the weighted-vote total of any candidate exceeds the threshold [i.e., while *any*(**f** > *t*_0_)], do the following:
    1.5.1 A weighted-vote total is *deficient* if it is less than *t*_0_. Every candidate whose weighted-vote total is not deficient is identified as an elected candidate. Set **nd** equal to *find*(**f** ≥ *t*_0_), which contains the indices of **f** at which **f** is not deficient. Note that 1 ≤ |**nd**| ≤ *n* if no rounding errors have occurred.
    1.5.2 If |**nd**| > *n*, then rounding errors have occurred at the limit of the computer’s numerical precision. Correct them by setting **f**(*y*) equal to *t*_0_ for every index, *y*, of **f** such that **f**(*y*) > *t*_0_. Then continue from Instruction 1.6.
    1.5.3 If |**nd**| = *n*, then return **c**(**nd**) as the vector whose elements identify the *n* election winners and exit APV.
    1.5.4 The portion of a candidate’s weighted-vote total above the threshold, *t*_0_, does not contribute to the candidate’s election and is, therefore, wasted unless redistributed to candidates whose weighted-vote totals are deficient to help fill the remaining *n* − |**nd**| > 0 unfilled positions. A weighted-vote total is *excessive* if it exceeds *t*_0_. Remove the excess weighted-vote totals from the candidates with excessive weighted-vote totals as follows: for each index, *y*, of **f** such that **f**(*y*) > *t*_0_, set **F**(:, *y*) equal to **F**(:, *y*) * *t*_0_/**f**(*y*).
    1.5.5 Transfer the excess weighted-vote totals to the candidates with deficient weighted-vote totals as follows: Set **d** equal to find(**f** < *t*_0_) which contains the indices of **f** at which **f** is deficient. For each row index, *x*, of **F**, set **F**(*x*, **d**) equal to
$$\left(1-{\left\|\text{F}\left(x,\text{nd}\right)\right\|}_{1}\right)*\text{pmf}\left[\text{F}\left(x,\text{d}\right)\right].$$
      Note that every row of **F** represents a pmf on the set of candidates represented by **c**, i.e., **F** equals *pmf*(**F**).
    1.5.6 Update **f** as follows: For each index, *y*, of **f** such that **f**(*y*) > *t*_0_, set **f**(*y*) equal to *t*_0_. And set **f**(**d**) equal to **w** * **F**(:, **d**). Note that **f** equals **w** * **F**.
  1.6 No excess candidate weighted-vote totals remain. After all excess candidate weighted-vote totals have been reallocated, a candidate is identified as *defeated* if the candidate cannot be elected without eliminating a candidate with a larger weighted-vote total. Identify and remove defeated candidates from **c** as follows:
    1.6.1 Let *T* be the largest element of **f** such that
$$T>{\left\|\text{f}\left(\text{f}<T\right)\right\|}_{1}.$$
      Note that no candidate in **c**(**f** < *T*) can be elected without eliminating one or more candidates in **c**(**f** ≥ *T*) because ‖**f**(**f** < *T*)‖_1_ < *T* ≤ *t*_0_ and |**c**(**f** ≥ *T*)| > *n*.
    1.6.2 If *any*(**f** < *T*), then remove the candidates in **c**(**f** < *T*) from **c** by setting **c**(**f** < *T*) equal to [](null). (Note that APV takes advantage of opportunities to eliminate multiple candidates simultaneously.)
    1.6.3 Otherwise, *T* is the smallest element of **f**, i.e., **c** equals **c**(**f** ≥ *T*). At least two elements of **f** equal *T* because, if not, the smallest element of **f**(**f** > *T*) satisfies the conditions of Instruction 1.6.1 above, contradicting *T*’s status as the largest such element of **f**. Set **Tindices** equal to find(**f** ≤ *T*), which contains the indices of **f** at which **f** equals *T*. Then randomly select a candidate from **c**(**Tindices**) and remove it from **c**.
2 Return **c** as the vector whose elements identify the election winners and exit APV.

This completes the APV algorithm. Note that the algorithm has two possible exit points: at Instructions 1.5.3 and 2. Also note that the algorithm does not make any approximations: all computations are exact to the limit of the computer’s numerical precision.

The next subsection, Notation reference, describes the mathematical notation used above. The Octave scripts listed in Subsection Octave scripts provide further algorithm details and optimizations [13]. Subsection Discussion discusses APV and provides an example of a possible APV ballot system that allows the voter to allocate support to candidates both individually and by party.

### Notation reference

Subsection APV description uses the following notation:

- Nonnegative functions on a set of candidates are represented by nonnegative row vectors whose column indices represent the candidates and whose value at each column index is the value of the function at the candidate represented by the column index.
- For every finite vector, **v**, |**v**| denotes the length (number of elements) of **v**.
- For every finite nonnegative vector, **v**,
$${\left\|\text{v}\right\|}_{1}$$
  denotes the 1-norm of **v**, i.e., the sum of the vector’s elements.
* denotes the matrix-multiplication operator.
- For every nonempty finite nonnegative row vector, **v**, *pmf*(**v**) denotes the pmf vector corresponding to **v**. Note that
$$\text{pmf}\left(\text{v}\right)=\left\{\begin{array}{cc} {{\left\|\text{v}\right\|}_{1}}^{-1}*\text{v} & \text{if}\;{\left\|\text{v}\right\|}_{1}>0\\ {{\left\|\text{e}\right\|}_{1}}^{-1}*\text{e} & \text{otherwise} \end{array}\right.,$$
  where **e** is the row vector whose length is |**v**| and every element’s value is one.
- In general, for every nonempty finite nonnegative matrix, **M**, *pmf*(**M**) denotes the matrix with the same number of rows as **M** such that, for each row, **r**, of **M**, the corresponding row of pmf(**M**) is pmf(**r**).
- For every vector, **a**, and finite vector, **b**, of **a**’s indices, **a**(**b**) denotes the vector of length |**b**| and of the same shape (row or column) as **a**, whose *j*th element, **a**(**b**)(*j*) is
$$\text{a}\left[\text{b}\left(j\right)\right].$$
- For every matrix, **M**, finite vector, **r**, of **M**’s row indices, and finite vector, **c**, of **M**’s column indices,
  - **M**(**r**,**c**) denotes the |**r**| by |**c**| matrix whose element **M**(**r**,**c**)(*x*,*y*) with row index *x* and column index *y* is
$$\text{M}\left[\text{r}\left(x\right),\text{c}\left(y\right)\right].$$
  - **M**(**r**,:) denotes **M**(**r**,**c**) when |**c**| is a column index of **M** and **c**(*y*) = *y* for every column index, *y*, of **M**.
  - **M**(:, **c**) denotes **M**(**r**,**c**) when |**r**| is a row index of **M** and **r**(*x*) = *x* for every row index, *x*, of **M**.
- For every logical vector, **b**,
  - find(**b**) denotes the vector obtained from a copy, **a**, of **b** by setting every element, **a**(*j*), of **a** whose value is “true” equal to *j* and deleting every element of **a** whose value is “false.”
  - any(**b**)’s value is “true” if **b** has an element equal to “true,” and is “false” otherwise.
- For every finite vector, **a**, and logical vector, **b**, such that |**b**| = |**a**|, **a**(**b**) denotes
$$\text{a}\left[\text{find}\left(\text{b}\right)\right].$$

### Octave scripts

APV was coded, tested, and refined, in the Octave scripting language [13]. An Octave script is a list of computer instructions in the Octave scripting language. The script is in a text file with a.m extension. An Octave interpreter reads script files and executes the computer instructions contained in them. Octave scripts that implement APV are given in this subsection along with the scripts used to run the examples of APV’s election performance given in Subsection Apportioned-voting examples of the next section. The Octave interpreter is freely available for download and use from https://www.octave.org/download.html and https://ftp.gnu.org/gnu/octave/. [13] is a detailed reference to the Octave scripting language and interpreter and is available from https://www.gnu.org/software/octave/doc/v9.2.0/ and the Documentation submenu of the Octave interpreter’s Help menu. The Octave and MATLAB^®^ scripting languages are closely related and most scripts are easily portable [13].

The following subsections list the Octave scripts used to implement the APV algorithm given in Subsection APV description and obtain the results of the examples of APV’s election performance given in Subsection Apportioned-voting examples [13]. Function apv, in Subsection Script for function apv, runs APV and returns numbers identifying the elected candidates. Two potentially-large inputs, **V** and **w**, of apv are prepared as Class ptr objects (see Subsection Script for object-class ptr) to reduce data storage requirements and run time. Function pmf, in Subsection Script for function pmf, is used internally by apv to convert nonnegative functions on sets of candidates to probability mass functions. The script in Subsection Script to run example 1 yields the results of Example 1. The script in Subsection Script to run example 2 yields the results of Example 2. Each script may be copied from this document, pasted into a new blank script in the Octave Editor, and saved as a file with the desired file name and “.m” extension. The script files may be in the same directory. The Octave interpreter is freely available for download and use from https://www.octave.org/download.html or https://ftp.gnu.org/gnu/octave/ [13].

#### Script for function apv

The Octave interpreter runs the following script for function apv from a text file named apv.m [13].

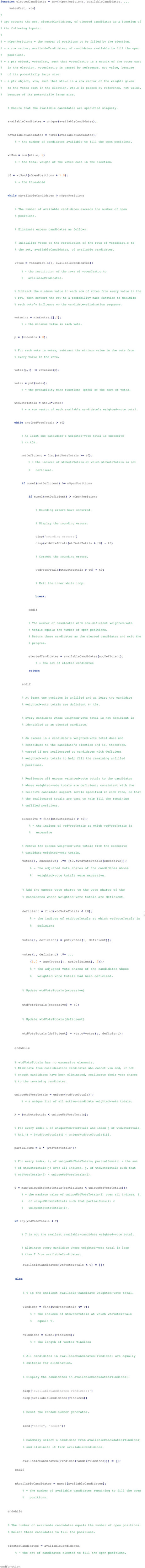


#### Script for object-class ptr

The following object-class script may be run by the Octave interpreter from a text file named ptr.m [13]:

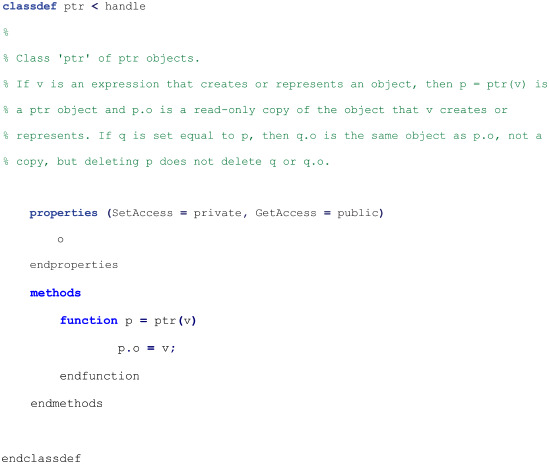


#### Script for function pmf

The following function script is used by the script for Function apv and may be run by the Octave interpreter from a text file named pmf.m [13]:

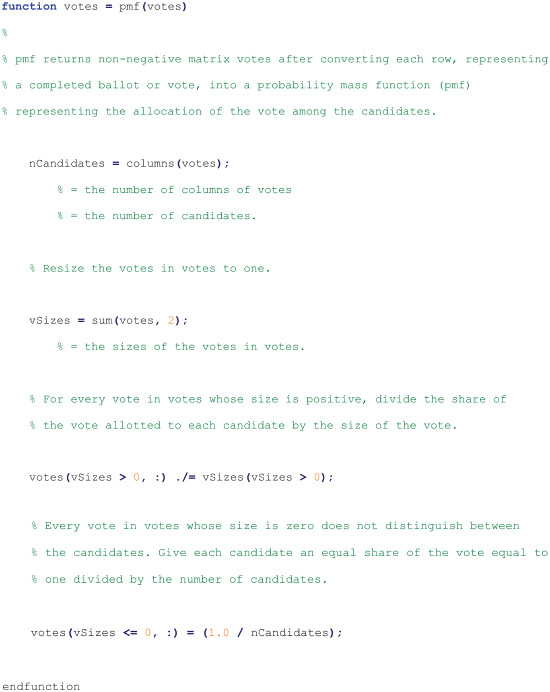


#### Script to run example 1

The following Octave script was run by Octave interpreter version 9.2.0 from a text file with a “.m” extension to obtain the results given in Subsection Example 1: proportional representation of Subsection Apportioned-voting examples of Section Results and discussion [13]:

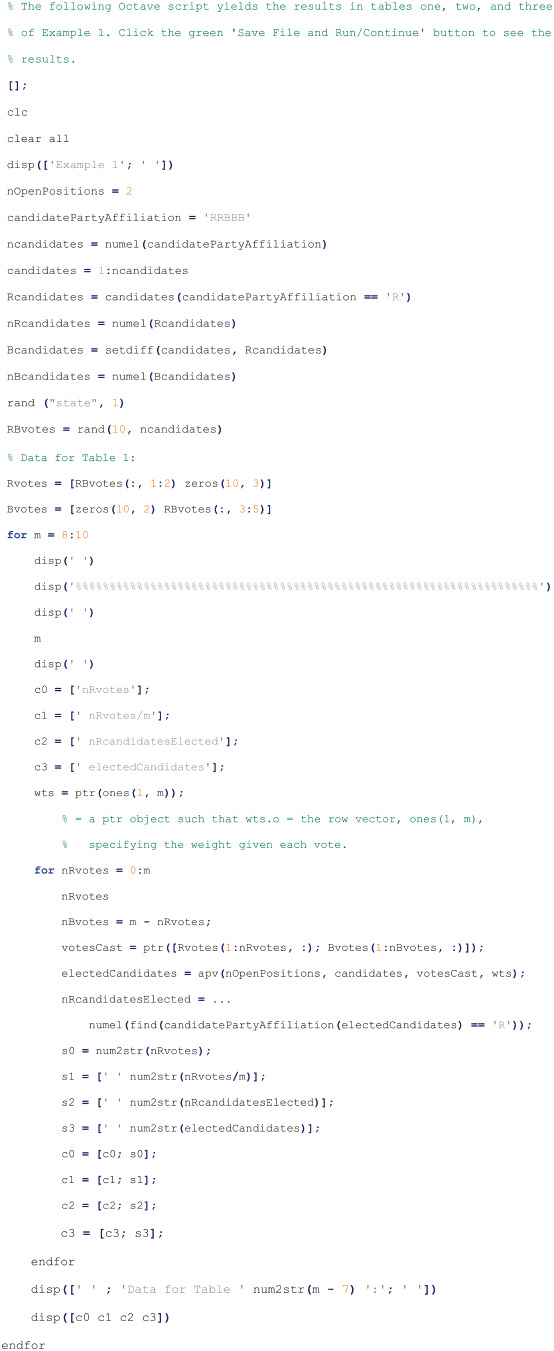


#### Script to run example 2

The following Octave script was run by Octave interpreter version 9.2.0 from a text file with a “.m” extension to obtain the results given in Subsection Example 2: efficiency of Subsection Apportioned-voting examples of Section Results and discussion [13]:

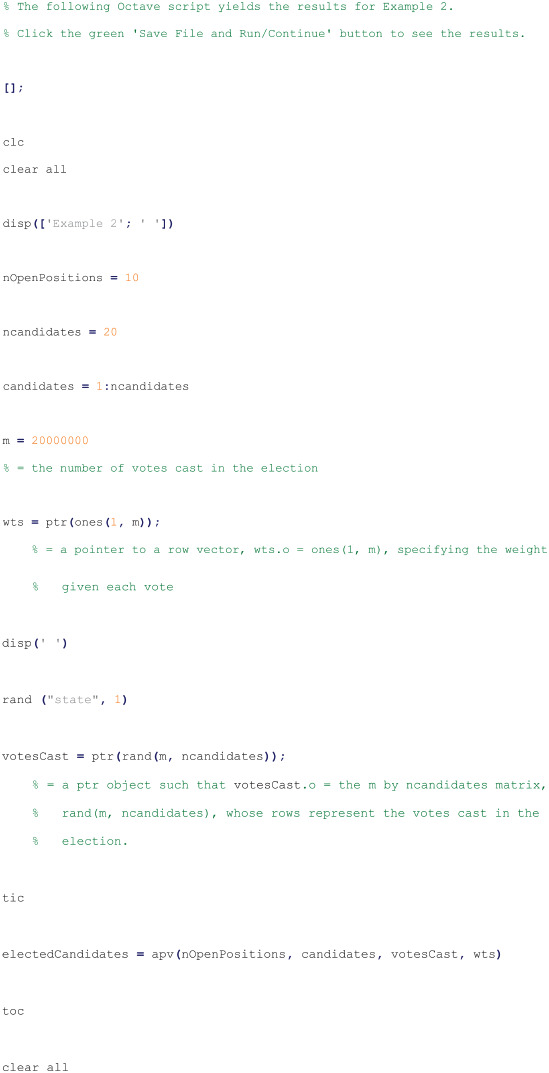


## Results and discussion

Subsection Apportioned-voting examples gives the results of applying APV to some election examples. The results illustrate how APV behaves in practice. Subsection Discussion discusses some of APV’s features and gives some suggestions for applying APV in practice, including an example of a possible ballot system that permits the allocation of support to candidates individually and by party.

### Apportioned-voting examples

This subsection gives election examples illustrating how APV behaves in practice.

The first example, Example 1: proportional representation, illustrates that APV provides proportional representation of voting blocs in election results. The results of two-party partisan bloc voting, as a function of the total number of votes cast and the number of votes cast for each party, are compared.

The second example, Example 2: efficiency, illustrates the efficiency of APV when applied to elections with large numbers of voters (20 million), candidates (20), and positions to be filled (10).

Subsection Octave scripts of Section Materials and methods gives listings of the Octave scripts used to obtain the example results. The Octave interpreter is freely available for download and use from https://www.octave.org/download.html and https://ftp.gnu.org/gnu/octave/ [13].

#### Example 1: Proportional representation

A partisan bloc vote is of the form, **V**(*x*,:), where, *x* is a row (vote) index of **V** and, for every column (candidate) index, *y*, of **V**, **V**(*x*, *y*) is positive if the *y*th column of **V** represents a candidate of a certain political party, and zero otherwise. Using the APV procedure, the following example compares the results of two-party partisan bloc voting, as a function of the total number of votes cast and the number of votes cast for each party.

Suppose the following:

- The number of positions to be filled by the election is *n* = 2.
- The vector of numeric identifiers of the candidates available to fill the positions is **c** = 1: 5 = [1 2 3 4 5].
- Candidates one and two represent the R party, and candidates three, four, and five represent the B party.
- The number of votes cast in the election is *m*.
- **RBvotes** is the 10-by-5 random-sample matrix of independent and uniformly-distributed pseudo-random numbers on the unit interval generated by first calling rand("state", 1) to initialize the Octave pseudo-random number generator “rand”; then setting **RBvotes** equal to *rand*(10,5).
- The *m* -by-5 matrix, **V**, of votes cast consists of the first *nRvotes* rows of matrix **Rvotes** and the first *nBvotes* = *m* − *nRvotes* rows of matrix **Bvotes**, where
  - **Rvotes** consists of the first two columns of **RBvotes** followed by three all-zero columns, and
  - **Bvotes** consists of two all-zero columns followed by the last three columns of **RBvotes**.
- The vote-weight vector, **w**, is the row vector with exactly *m* elements each of which equals one.
- The number of R-party candidates elected is *nRElected*.

Then the following runs of the APV procedure, *apv*(*n*, **c**, **V**, **w**), were made and results obtained:

Table 1 gives the results of applying the APV procedure to the election for *nRvotes* = 0: 8 when *m* = 8.

**Table 1:** Results for Example 1’s eight-vote election.

*nRvotes*	*nRvotes*/8	*nRElected*	Candidates Elected	
0	0	0	4	5
1	0.125	0	4	5
2	0.25	0	3	5
3	0.375	1	2	5
4	0.5	1	1	5
5	0.625	1	1	5
6	0.75	2	1	2
7	0.875	2	1	2
8	1	2	1	2

Table 2 gives the results of applying the APV procedure to the election for *nRvotes* = 0: 9 when *m* = 9.

**Table 2:** Results for Example 1’s nine-vote election.

*nRvotes*	*nRvotes*/9	*nRElected*	Candidates Elected	
0	0	0	4	5
1	1/9	0	4	5
2	2/9	0	3	5
3	1/3	1	2	5
4	4/9	1	1	5
5	5/9	1	1	5
6	2/3	2	1	2
7	7/9	2	1	2
8	8/9	2	1	2
9	1	2	1	2

Table 3 gives the results of applying the APV procedure to the election for *nRvotes* = 0: 10 when *m* = 10.

**Table 3:** Results for Example 1’s ten-vote election.

*nRvotes*	*nRvotes*/10	*nRElected*	Candidates Elected	
0	0.0	0	3	5
1	0.1	0	4	5
2	0.2	0	4	5
3	0.3	0	3	5
4	0.4	1	1	5
5	0.5	1	1	5
6	0.6	1	1	5
7	0.7	2	1	2
8	0.8	2	1	2
9	0.9	2	1	2
10	1.0	2	1	2

All election results were nonrandom because Instruction 1.6.3 of the APV procedure was not executed.

The candidates elected are the first two to be identified as elected, i.e., given nondeficient weighted-vote totals.

When the proportion, *nRvotes*/*m*, of the votes cast for the R party is below 1/3, all candidates elected belong to the B party because *nRvotes* < *m*/3 = *m*/(*n* + 1) = *t*_0_ = the minimum possible weighted-vote total of an elected candidate, so, R-party votes are not sufficient to elect any candidate while B-party votes can elect two (2*t*_0_ < *nBvotes*).

When *nRvotes*/*m* is strictly between 1/3 and 2/3, one candidate from each party is elected because *t*_0_ < *nRvotes* < 2*t*_0_ and *t*_0_ < *nBvotes* < 2*t*_0_, so, each party can elect one, but not two, candidates.

When *nRvotes*/*m* exceeds 2/3, all candidates elected belong to the R party because B-party votes are not sufficient to elect any candidate (*nBvotes* < *t*_0_) while R-party votes can elect two (2*t*_0_ < *nRvotes*).

When *nRvotes*/*m* equals 1/3, at least one candidate elected will belong to the B party because R-party votes can elect no more than one candidate (*nRvotes* = *t*_0_) while B-party votes can elect up to two candidates (*nBvotes* = 2*t*_0_). Similarly, when *nRvotes*/*m* equals 2/3, at least one candidate elected will belong to the R party because B-party votes can elect no more than one candidate (*nBvotes* = *t*_0_) while R-party votes can elect up to two candidates (*nRvotes* = 2*t*_0_). In these two cases, the conditions on the votes-cast matrix, **V**, that determine whether or not the elected candidates belong to the same party and whether or not this outcome is random have not been identified.

#### Example 2: efficiency

The APV procedure was run with the following election parameter values:

- The number of positions to be filled by the election is *n* = 10.
- The vector of numeric identifiers for the candidates on the ballot is ***c*** = 1: 20 containing the first 20 positive integers.
- The number of votes cast in the election is *m* = 20 million.
- The matrix, **V**, of votes cast is the *m* -by-20 random-sample matrix of independent and uniformly-distributed pseudo-random numbers on the unit interval generated by first calling rand("state", 1) to initialize the Octave pseudo-random number generator “rand”; then setting **V** equal to rand(*m*, 20) [13]. Note that **V** has 400 million elements.
- The vote-weight vector, **w**, is the row vector of length *m* all of whose entries are one. Note that **w** has 20 million elements.

The following results were obtained:

- The vector, *apv*(*n*, **c**, **V**, **w**), of candidates elected was [1 2 4 5 9 13 15 16 17 20].
- The election results were nonrandom because Instruction 1.6.3 of the APV procedure was not executed.
- The run time of the procedure was 148.702 seconds (about 2.5 minutes) on a home desktop PC running 64-bit Windows 10 with 16.0 GB (14.9 GB usable) of installed RAM. Note that the procedure’s run time is not constant, but depends on the computer’s hardware, operating system, other processes running on the computer, and amount of free memory available on the computer. For data sets exceeding 450 million elements, it is likely that the amount of installed RAM would have to exceed 16 GB, and that, for data sets exceeding 2 billion elements, the amount of installed RAM would have to exceed 32 GB and the version of Octave used would have to be the version that can perform 64-bit linear algebra for large (more than 2 billion-element) data sets [13].

### Discussion

This section discusses various features, capabilities, and benefits of APV.

APV assumes that, on each submitted ballot (vote cast), the voter has assigned each candidate a nonnegative number representing the voter’s level of support for or rating of the candidate relative to the other candidates. Under this assumption, if a candidate is removed from the ballot, there is no need to change already submitted ballots: the ballots continue to satisfy the assumption after the candidate is removed (e.g., see Instruction 1.1 of the APV procedure).

APV can accept data from any completed ballot that can be interpreted as representing a nonnegative level-of-support function on the set of candidates. For example, APV could accept data from single-vote plurality, approval-voting, List-PR, approximate rank-voting or STV, CV, or grade-voting election ballots [14, 15].

A ballot designed specifically for APV might have the following form:

- The voter could use a stand-alone computer app or script to complete a ballot and print an official paper copy of the completed ballot. The app or script would permit the voter to do the following:
  - Specify a nonnegative number to represent a level of support.
  - Click on or tap a candidate’s name to automatically assign the specified level of support to the candidate and, if the candidate represents a political party, update the level of support assigned to the party (note that a party’s level of support is the sum of the levels of support assigned to the party’s candidates).
  - Click on or tap a party’s name to automatically assign the specified level of support to the party and update the levels of support assigned to the party’s candidates by normalizing them so that they sum to one; then, multiplying them by the specified level of support.
  - Undo the last action if necessary.
  - Repeat these actions until satisfied with the result.
  The initial and default level of support assigned to every candidate and party is zero. A bar chart could be used as a visual aid. A candidate’s or party’s name and level of support would be displayed along each bar. The length of the bar would be proportional to the level of support displayed along the bar. When satisfied with the result, the voter would print an official paper copy of the completed ballot.
- To cast a vote, the voter would submit the completed paper ballot at a polling place. A scan of the paper ballot would supply the data needed by APV. APV uses only candidate data.

Note that each party has a list of candidates running in the election. The parties’ candidate lists must not overlap. Every candidate must represent at most one party. A voter can assign relative levels of support to candidates either directly or by supporting their political parties if any. A party’s support is distributed among its candidates so that the party’s level of support equals the sum of the levels of support assigned to its candidates. APV determines how many and which candidates are elected from each party’s list of candidates.

The weights that apply to APV’s votes are not voter inputs to APV, but are determined by the authorities governing the election.

An important advantage of APV over other voting systems, except grading, is that it offers voters a greater range of options for expressing candidate support [14, 15]. APV and grading can offer the same range of options for expressing candidate support, but APV and grading process their inputs differently [15]. See [15], the description of a possible APV ballot above, and Subsection APV description.

Other problems with voting systems that APV can correct include the following:

Grading does not weight votes equally when computing candidate vote totals because it does not normalize votes before adding them [15]. APV normalizes votes automatically before computing weighted-vote totals.

CV requires that each vote report relative support for candidates with the sum of its numbers equal to its allowed weight [4–6]. This puts an added burden on the voter. APV only requires voters to grade or rate the candidates on whatever nonnegative scale the voter desires. It does not require voters to distribute a fixed amount of support among the candidates.

Unlike APV, CV does not identify defeated candidates for elimination and reallocate their support to the remaining candidates and, if the election is to fill more than one position, CV does not reallocate excess support from elected candidates to candidates that need it to fill remaining unfilled positions [4–6].

Under STV, voters complete their ballots by ranking the candidates on the ballot by preference [7–10]. A candidate ranking cannot represent a nontrivial level-of-support function on the set of candidates. Professor Nicolaus Tideman, who has reviewed this paper, observed that “apportioned voting becomes equivalent to STV in the limit as voters allocate their votes in larger and larger sequential proportions: One … for the first candidate, one thousandth … for the second candidate, one millionth … for the third candidate, and so on.” Conceptually, APV can approximate STV as closely as desired. Therefore, APV is capable of offering voters a much greater range of options for expressing candidate support than STV.

Voter inputs to STV can be derived from voter inputs to APV, but not vice versa. STV’s voter inputs provide STV with only the directions of each-voter’s preferences, not their relative strengths. APV’s voter inputs provide APV with both the directions and relative strengths of each-voter’s preferences. APV bases its election results on more information about voter preferences than STV.

STV requires that each voter rank the candidates. To rank the candidates, the voter must sort them by preference. Sorting *k* candidates is of order *k log k* or *k*^2^ hard, depending on the sorting method. APV requires that each voter grade or rate the candidates. Rating *k* candidates is of order *k* hard because rating each candidate requires only that the voter compare the candidate to one previously-rated candidate whose rating is positive, if one exists. APV does not require that a voter sort the candidates.

Under APV, the voters determine how many and which of each party’s candidates are elected. Under List PR, the voters determine how many of each party’s candidates are elected, but each party may determine which of its candidates are elected.

APV takes into account every-voter’s secondary preferences when computing candidate vote totals because each vote may distribute the voter’s support among the candidates and, when computing candidate vote totals, APV uses a quota or threshold system and a process of candidate elimination that reallocate levels of voter support from candidates that do not need them to candidates that do until all open positions are filled. List PR doesn’t take into account any voter’s secondary preferences when computing party or candidate vote totals because each vote must give all the voter’s support to one party or candidate.

APV eliminates the need for primary and runoff elections. All candidates can participate directly in the general election and APV will fill all open positions whenever there are enough candidates to fill them.

Voting-district boundaries are often politically manipulated to affect election outcomes. APV provides the option of consolidating voting districts. Periodic at-large elections that provide proportional representation can help eliminate the need for voting districts. For example, suppose that a certain legislative body has 100 members and that an at-large APV election is held annually to fill 20 seats. Then each seat would be up for election every five years without the need to partition the seats and voters into voting districts. At-large APV elections can also be expected to improve the legislative process and its output because they hold each lawmaker accountable to every voter affected by the lawmaker’s performance as a legislator. Regional interests could be represented by political parties with regionally-restricted memberships. Like every other party, they would provide candidates to compete in general elections. Regional governments and associations might perform this function. If districts are required, they can be managed in the same way they are now. The voting systems of current general elections could be replaced with APV, permitting the elimination of primary and runoff elections.

The use of subjective and politically-biased criteria to qualify candidates for the ballot limits the political diversity of the candidates on the ballot. The political diversity of the candidates on the ballot can be expected to increase with the number of candidates on the ballot and the objectivity of the criteria used to qualify them for the ballot. Examples of objective criteria that might be used to qualify candidates for the ballot include the following:

- Level of support for the candidate in recent public opinion polls.
- Number of recent individual ballot-access-petition signers in support of the candidate.
- Number of recent individual donors to the candidate.
- Size of the candidate’s constituency if the candidate holds an elective office.
- Level of public support for the candidate’s political party if the candidate represents a political party.

A legislature could use APV in the legislative process to select a proposal from among multiple legislative proposals; then use an up-or-down vote to determine whether or not to approve (pass) the selected proposal. Effectively, the goal is to determine which proposal has the most support and, then, determine whether this support is sufficient to pass it. For example, this process might be used to select and pass a budget proposal. The proposals need not be related to one another.
